# Effect of Fufang Biejia Ruangan Tablet on lowering biochemical and virological parameters of hepatic fibrosis in patients with chronic hepatitis B: Protocol for a systematic review and meta-analysis of randomized controlled trials and cohort studies

**DOI:** 10.1097/MD.0000000000015297

**Published:** 2019-04-26

**Authors:** Chaoyuan Huang, Danting Shen, Shuning Sun, Yuancheng Huang, Yijun Xin, Hu Luo, Yinzhen Chen, Zipu Zhou, Fengbin Liu, Xinlin Chen

**Affiliations:** a The First Clinical College, Guangzhou University of Chinese Medicine; b Department of Gastroenterology, The First Affiliated Hospital of Guangzhou University of Chinese Medicine; c School of Basic Medical Science, Guangzhou University of Chinese Medicine, Guangzhou, China.

**Keywords:** biochemical parameters, Fufang Biejia Ruangan Tablet, hepatic fibrosis, meta-analysis, protocol, systematic review, virological parameters

## Abstract

**Background:**

Liver cirrhosis is one of the end-stage chronic liver diseases. Individuals with chronic hepatitis B (CHB) are at an increased risk of developing liver cirrhosis. Practice guidelines underline that Nucleos(t)ide analogs (NAs) should be the first-line treatment for hepatitis B virus (HBV)-related cirrhosis. However, prolonged use of NAs may lead to drug resistance and kidney impair and does not reverse the fibrosis of liver. Fufang Biejia Ruangan Tablet (RGT), as a traditional Chinese medicine (TCM), has been proved to be effective in the treatment of liver fibrosis. Hence, we will perform meta-analysis in order to evaluate the efficacy and safety of RGT in the treatment of hepatic fibrosis in patients with CHB.

**Methods:**

To search for relative literatures up to February 2019 by computer from the following databases: PubMed, Embase, Cochrane Library, Chinese National Knowledge Infrastructure, Chinese Science and Technology Periodicals Database, Chinese BioMedical Database and Wanfang Data. Included criteria are randomized controlled trials and cohort studies of hepatic fibrosis in patients with CHB treated by RGT. The primary outcome measures include biochemical and virological parameters. We will use Stata 13.0 software for data synthesis, sensitivity analysis, meta regression, subgroup analysis, and risk of bias assessment. The reporting bias will be assessed by a funnel plot and the funnel plot symmetries will be evaluated by Begg and Egger tests. We will use the Grading of Recommendations Assessment, Development and Evaluation system to assess the quality of evidence.

**Results:**

This systematic review will provide a synthesis of RGT for hepatic fibrosis in patients with CHB from various evaluation aspects including biochemical and virological parameters, HBV DNA levels HBeAg status and seroconversion, adverse events incidence.

**Conclusion:**

The systematic review will provide evidence to assess the efficacy and safety of RGT in the treatment of hepatic fibrosis in patients with CHB.

**PROSPERO registration number:**

ROSPERO CRD 42018095122.

## 1 Introduction

### 1.1 Description of the background

Characterized by fibrosis, structural abnormal nodules, and the damaged hepatic lobule,^[1]^ cirrhosis is one of the end-stage chronic liver diseases.^[2]^ Individuals with chronic hepatitis B (CHB) are at an increased risk of developing liver cirrhosis.^[3]^ According to recent estimates of the World Health Organization, cirrhosis (720,000 deaths) accounts for more deaths than hepatocellular carcinoma (470,000 deaths) in the long-term complications of hepatitis B virus (HBV) infections. In 2015, it was projected that 257 million persons, or 3.5% of the population, were living with chronic HBV infection in the world, and 1.34 million deaths would occur owing to viral hepatitis.^[2]^ Moreover, the number of deaths will continue to increase unless people with HBV infection are diagnosed and treated. With the ever increasing mortality, cirrhosis is highly regarded as a public health problem worldwide.

### 1.2 Description of the intervention

Practice guidelines underline that oral antiviral therapy should be the first-line treatment for HBV-related cirrhosis. Nucleos(t)ide analogs (NAs) are recommended for such patients because they can inhibit HBV replication, which help block liver fibrosis and prevent the development of hepatocellular carcinoma (HCC).^[1]^ However, prolonged use of NAs may lead to drug resistance and kidney impair, the most importantly, eradication or suppression of HBV does not reverse the fibrosis of liver and eliminate the risk of occurrence of HCC.^[4]^ Fufang Biejia Ruangan Tablet (RGT), as a traditional Chinese medicine (TCM), has been proved to be effective in the treatment of liver fibrosis. It is the first antifibrotic herb which has passed the Good Clinical Practice (GCP) certification by the Chinese Food and Drug Administration (CFDA).^[5]^ Studies have revealed that the combination therapy with RGT and oral NUCs was more effective than oral NAs alone for treating HBV-related cirrhosis.^[6,7]^ Current systematic reviews and meta-analyses on the effect of the combination therapy with RGT and NAs for HBV-related cirrhosis are not registered and with limited quality and quantity of included studies. To address this issue, we plan to conduct a systematic review and meta-analysis to evaluate current evidence on the effects of the combination therapy of RGT plus NAs in treating HBV-related cirrhosis.

## 2 Methods

### 2.1 Inclusion criteria for study selection

#### 2.1.1 Types of studies

All randomized controlled trials and cohort studies that evaluate the safety and effect of combination therapy of RGT plus entecavir (ETV) on the liver cirrhosis of chronic hepatitis B will be incorporated into our study.

#### 2.1.2 Types of patients

Patients were diagnosed cirrhosis or hepatic fibrosis that caused by HBV.

#### 2.1.3 Types of interventions

Patients from control group received the routine oral antiviral therapy, in this study, we will solely include ETV as it has been proved to be the first line regimen. The intervention group will be treated by RGT and ETV.

#### 2.1.4 Types of outcome measures

##### 2.1.4.1 The primary outcome measures will include biochemical parameters that could monitor the liver function and liver fibrosis status

ALT, AST, TBiL, HA, LN, PCIII, and IV-C.

##### 2.1.4.2 The secondary outcome measures will be virological indicators such as HBV DNA levels HBeAg status and seroconversion

Adverse events will be comprised.

### 2.2 Search methods for the identification of studies

The articles from inception to November 2017 were searched in the following databases: PubMed, Embase, Cochrane Library, Chinese National Knowledge Infrastructure (CNKI), Wanfang Data, Chinese Biomedical Literature Database and Chinese Science (CBM) and Chinese Science and Technology Periodical Database (VIP). The keywords used to search include “hepatitis B virus,” “chronic hepatitis B,” “hepatitis B,” “Liver Cirrhosis,” “hepatic fibrosis,” “biejiaruangan,” and “biejia ruangan.”

The search strategy will be “hepatitis B virus” OR “chronic hepatitis B” OR “hepatitis B” AND “Liver Cirrhosis” OR “Hepatic Fibrosis”’ AND “biejiaruangan” OR “biejia ruangan.” Chinese translations of these terms will be used for the Chinese databases.

**Figure 1:**
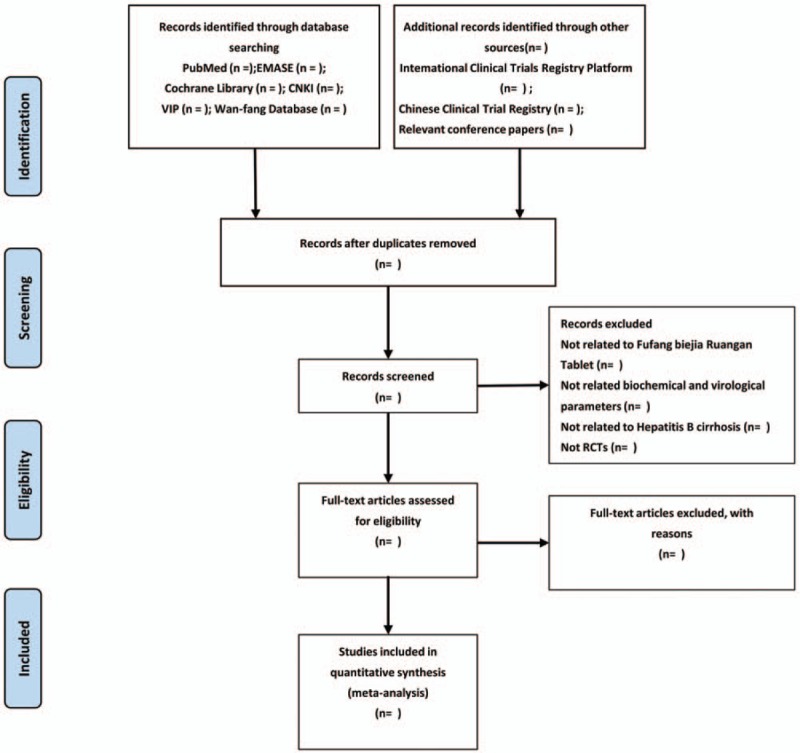
Flow diagram of study selection process.

Two researchers (SS and DS) will evaluate the full texts independently and identify the potential eligible studies. If there is an objection about the articles between the 2 researchers, objections will be delivered to the third researcher (CH) and final decision will be made by him. A Preferred Reporting Items for Systematic Reviews and Meta-Analyses (PRISMA) flowchart will be created to show the number of articles identified, screened, included and excluded, reasons for exclusion and to confirm eligible studies. The study selection process will be described in a PRISMA flowchart. (http://www.prisma-statement.org) (Fig. 1)

### 2.3 Data collection and analysis

#### 2.3.1 Selection of studies

The literatures will be excluded when the absences of both the primary and secondary outcome measures were happened. Two researchers (SS and DS) will narrow down the potential eligible literatures based on the PICOS standard by scanning the titles and abstracts. Also then full texts reading will be completed to select the final literatures that meet the included criteria. Selected literatures management and duplication removal will be administrated via Endnote V.X7.

#### 2.3.2 Data extraction and management

A self-made data extraction form will be used to extract data, including authorship, publication year, study type, the number of participants, interventions, and outcome measures. Two researchers (SS and DS) will extract data separately. If there are disagreements, they will settle through discussion. If necessary, the divergence will be discussed with the third author (CH).

#### 2.3.3 Addressing missing data or unclear measurement scales

We will contact the authors by email for further information about the articles while there are missing data or unclear measurement scales. If sufficient information cannot be reached through this way, we will analyze the available data. The potential impact of the missing data will be discussed in Section 4.

#### 2.3.4 Assessment of risk of bias in included studies

Risk of bias in all included articles will be evaluated on the basis of the Cochrane Handbook for Systematic Reviews of Interventions. The following sources of bias will be considered in this assessment tool: random sequence generation, allocation concealment, blinding of outcome assessments, incomplete outcome data, and selective outcome reporting. The risk level will be classified as low, high, or unclear (unclear or unknown risk of bias).

#### 2.3.5 Measures of treatment effect

The dichotomous outcomes will be expressed by relative risk (RR) or odds ratio (OR) with 95% confidence interval (CI). For continuous outcomes, mean difference (MD) with 95% CI will be presented if the outcome measures of all studies are based on the same unit of measurement, otherwise, standardized mean difference (SMD) with 95% CI will be presented for analysis.

#### 2.3.6 Data synthesis and analyses

Stata 13.0 will be used for synthesis and analysis of the data. Heterogeneity of included studies will be evaluated in the use of the *Q* and *I*^2^ test statistics. For the *Q* statistic, *P < *.05 will be considered as revealing significant variation. For the *I*^2^ statistic, *I*^2^ < 50% manifests the heterogeneity was acceptable and *I*^2^ > 50% indicates strong heterogeneity. If there is no heterogeneity among studies, fixed effects models will be adopted, otherwise, random effects models will be used.

#### 2.3.7 Additional analyses

Sensitivity analysis, meta-regression, and subgroup analysis might be conducted if there are potential sources of heterogeneity. Qualitative synthesis will be performed if data extraction is insufficient.

#### 2.3.8 Assessment of publication biases

A funnel plot will be established to assess the publication bias of the included studies. Begg and Egger tests will be used to estimate the symmetry of funnel plot.

#### 2.3.9 Quality of evidence

The Grading of Recommendations Assessment, Development, and Evaluation approach will be the tool of the evidence quality assessment. Limitations of the study, inconsistencies, indirect evidence, inaccuracies, and publication bias will draw the attention of researchers. Four levels of evidence quality will be used: high, moderate, low or very low.

## 3 Ethics and dissemination

We used the PRISMA-P checklist when writing our report.^[8]^ This study aims at providing available evidence for the safety of RGT and its effect on biochemical and virological parameters in the treatment of cirrhosis or hepatic fibrosis that caused by HBV. Ethical approval will not be demanded since there is no participant privacy involved.

## 4 Discussion

HBV infection, as one of the most common chronic viral infections in the world, will cause 40% of men and15% of women who have been infected to die of liver cirrhosis or hepatocellular carcinoma.^[9]^ When the prevalence of HBV infection is as high as 8% or above in one area (e.g., such as China, most of Pacific Islands and East Asia) the individuals who live in these areas have a high risk of developing complications of cirrhosis and hepatocelluar carcinoma.^[10]^

Treatment of CHB has dramatically improved over the past 10 decades. In 2008, NAs (e.g., lamivudine, adefovir, ETV, and telbivudine) were approved for the treatment of CHB in Europe and the USA.^[11]^ However, first-line NAs (e.g., ETV or tenofovir) has been preferred over other NAs in the latest updated guidance due to their higher potency and high resistance barrier.^[12]^ ETV, as the earliest NAs in the list of preferred HBV therapies, had been extensively used worldwide.^[12]^ Although ETV could achieve better biochemical and virological responses in CHB patients, it only delays the process of cirrhosis but not reverses or improves the state of cirrhosis.^[13]^ Therefore, current advances have demonstrated that the combination of TCM and NAs could have a better effect than NAs monotherapy on blocking and reversing the development of hepatic fibrosis or cirrhosis in patients with CHB.

RGT, as one of the Chinese patent drugs that is widely used in China for the adjuvant therapy of hepatic fibrosis or cirrhosis, has been reported repeatedly to be effective in improving the liver function and liver fibrosis status. With the main components of turtle shell (Carapax trionycis), zedoray rhizome (Rhizoma curcumae), peony root (Radix Paeoniae rubra), Angelica sinensis (Radix Angelica sinensis), pseudo-ginseng (Radix notoginseng), campanumaea pilosula (Radix codonopsis), Astragalus (Radix astragali), dried human placenta (Placenta hominis), plant worms (Cordyceps sinensis), Baphicacanthus root (Radix isatidis), and farsythio (Fructus forsythia),

RGT has the Chinese medical effects of softening hard masses (visible or latent stiff substances in human body such as tumor, cirrhosis or foreign body sensation in laryngeal) and removing stagnation, dissolving blood stasis and detoxification, reinforcing qi (invisible flowing substances in human body that preserves life and promotes blood circulation in TCM theory) and nourishing blood. Deng et al^[14]^ suggested that RGT may against hepatic fibrosis by reducing oxidative stress, inhibiting collagen fibrillation, reversing hepatic stellate cell activation and inhibiting the expression of hepatic growth factor.

Even though a mass of researches have indicated the remarkable effect of RGT in treating cirrhosis patients with CHB, the evidence that RGT can lower the biochemical and virological parameters are still vitally insufficient. With the purpose of systematically assessing the effect of RGT on biochemical and virological parameters in hepatic fibrosis or cirrhosis in patients with CHB, we aim to include adequate researches for the meta-analysis to ensure assertive evidence. We expect to reveal that RGT has a more positive effect on the patients with hepatic fibrosis or cirrhosis. The results of this review may help to provide reliable evidence for establishing a better regimen for prevention of hepatic fibrosis or cirrhosis.

## Author contributions

**Data curation:** Danting Shen, Shuning Sun, Yijun Xin, Hu Luo.

**Formal analysis:** Chaoyuan Huang, Danting Shen, Shuning Sun, Yijun Xin.

**Funding acquisition:** Fengbin Liu.

**Investigation:** Yuancheng Huang, Yinzhen Chen, Zipu Zhou.

**Methodology:** Chaoyuan Huang, Xinlin Chen.

**Project administration:** Chaoyuan Huang.

**Resources:** Chaoyuan Huang.

**Software:** Chaoyuan Huang, Danting Shen, Shuning Sun, Yijun Xin.

**Supervision:** Fengbin Liu.

**Validation:** Fengbin Liu.

**Writing – original draft:** Chaoyuan Huang.

**Writing – review & editing:** Chaoyuan Huang.
